# Conserved *cis*-regulatory regions in a large genomic landscape control SHH and BMP-regulated *Gremlin1* expression in mouse limb buds

**DOI:** 10.1186/1471-213X-12-23

**Published:** 2012-08-13

**Authors:** Aimée Zuniga, Frédéric Laurent, Javier Lopez-Rios, Christian Klasen, Nicolas Matt, Rolf Zeller

**Affiliations:** 1 Developmental Genetics, Department of Biomedicine, University of Basel, Mattenstrasse 28, CH-4058, Basel, Switzerland; 2 Transgenic Service, EMBL, Meyerhofstrasse 1, 69012, Heidelberg, Germany; 3 Present address: Max-Delbrueck-Centrum Berlin, Robert Roessle-Strasse 10, 13215, Berlin, Germany; 4 Present address: Université de Strasbourg, Institut de Biologie Cellulaire et Moléculaire, UPR 9022 CNRS, 15 rue René Descartes, 67084, Strasbourg, France

## Abstract

**Background:**

Mouse limb bud is a prime model to study the regulatory interactions that control vertebrate organogenesis. Major aspects of limb bud development are controlled by feedback loops that define a self-regulatory signalling system. The SHH/GREM1/AER-FGF feedback loop forms the core of this signalling system that operates between the posterior mesenchymal organiser and the ectodermal signalling centre. The BMP antagonist Gremlin1 (GREM1) is a critical node in this system, whose dynamic expression is controlled by BMP, SHH, and FGF signalling and key to normal progression of limb bud development. Previous analysis identified a distant *cis*-regulatory landscape within the neighbouring *Formin1* (*Fmn1*) locus that is required for *Grem1* expression, reminiscent of the genomic landscapes controlling *HoxD* and *Shh* expression in limb buds.

**Results:**

Three highly conserved regions (HMCO1-3) were identified within the previously defined critical genomic region and tested for their ability to regulate *Grem1* expression in mouse limb buds. Using a combination of BAC and conventional transgenic approaches, a 9 kb region located ~70 kb downstream of the *Grem1* transcription unit was identified. This region, termed *Grem1 Regulatory Sequence 1* (*GRS1*), is able to recapitulate major aspects of *Grem1* expression, as it drives expression of a *LacZ* reporter into the posterior and, to a lesser extent, in the distal-anterior mesenchyme. Crossing the *GRS1* transgene into embryos with alterations in the SHH and BMP pathways established that *GRS1* depends on SHH and is modulated by BMP signalling, i.e. integrates inputs from these pathways. Chromatin immunoprecipitation revealed interaction of endogenous GLI3 proteins with the core *cis*-regulatory elements in the *GRS1* region. As GLI3 is a mediator of SHH signal transduction, these results indicated that SHH directly controls *Grem1* expression through the *GRS1* region. Finally, all *cis*-regulatory regions within the *Grem1* genomic landscape locate to the DNAse I hypersensitive sites identified in this genomic region by the ENCODE consortium.

**Conclusions:**

This study establishes that distant *cis*-regulatory regions scattered through a larger genomic landscape control the highly dynamic expression of *Grem1*, which is key to normal progression of mouse limb bud development.

## Background

During embryonic development, the spatio-temporal expression of transcriptional regulators and morphogenetic signals is tightly controlled. In fact, different types of congenital malformations are caused by mutations affecting specific *cis*-regulatory and non-coding genomic regions that alter the expression of genes or gene clusters in specific tissues (reviewed in ref. [1-3]). Genome-wide functional mapping revealed many of the large genomic landscapes that control the expression of developmental regulator genes in a global and tissue-specific manner [4]. This study and others reveal that the *cis*-regulatory regions controlling gene expression in a particular tissue are often located several hundred kilobases (kb) or even megabases (Mb) up- or downstream of the transcriptional start sites in other loci, and may control the expression of several genes [5-7]. Recently, it has been shown how multiple *cis*-regulatory regions interact to control the expression of the 5’*HoxD* gene complex in the presumptive digit territory. The transcriptionally active part of the *5’HoxD* gene cluster forms a so-called regulatory archipelago in which dispersed *cis*-regulatory elements co-operate to control gene expression in the distal limb bud by interacting with proximal promoters over large distances [8]. In fact, regulatory landscapes with distant and dispersed *cis*-regulatory regions seem to be a recurring theme in the dynamic spatio-temporal regulation of genes that are essential for limb bud development (reviewed in ref. [2,9,10], see also below).

Vertebrate limb bud development is controlled by interactions between two main signalling centres, the apical ectodermal ridge (AER) and the zone of polarizing activity (ZPA) located in the posterior limb mesenchyme. These two signalling centres interact as part of a self-regulatory feedback signalling system involving several signalling pathways [11]. In particular, ZPA cells produce the Sonic Hedgehog (SHH) signal, which together with GREMLIN1-mediated antagonism of Bone Morphogenetic Proteins (BMPs) in the posterior-distal limb bud mesenchyme propagates Fibroblast Growth Factor (FGF) signalling in the AER [11-14]. This SHH/GREM1/AER-FGF feedback-signalling loop promotes distal progression of limb bud outgrowth and formation of the autopod which gives rise to carpals and digit rays (reviewed in ref. [15]). SHH expression in the posterior limb bud mesenchyme is controlled by a *cis*-regulatory region located about 800 kb upstream of the transcriptional start site. Deletion of this ZPA regulatory region (ZRS) results in loss of distal limb structures [16], while point mutations within the ZRS result in anterior ectopic *Shh* expression and duplications of the thumb and/or anterior digits in different species [17,18]. In cells actively transcribing *Shh*, the ZRS loops out of its chromosomal territory to the *Shh* transcription unit, which reveals how distant *cis*-regulatory elements control gene expression [19]. Recently, several transcriptional regulators have been identified that control *Shh* expression by directly interacting with the distant ZRS region. These include HOX proteins, the bHLH transcription factor HAND2, and ETS transcription factors, providing a glimpse of the complex transcriptional regulation of *Shh* in the posterior limb bud mesenchyme [20-22].

SHH signalling is transduced by the GLI family of transcriptional regulators and inhibits the constitutive processing of the full-length GLI3 activator to its repressor form GLI3R (see e.g. ref. [23]). Of the three GLI transcription factors expressed during limb bud development, only *Gli3* is essential on its own (see e.g. ref. [24]). The inactivation of *Gli3* alters morphogenetic feedback signalling and results in formation of additional anterior digits [23,25-27]. In particular, GLI3 restrains the proliferation of mesenchymal progenitors in the anterior limb bud mesenchyme and promotes initiation of digit ray chondrogenesis by directly repressing *Grem1* expression during handplate formation [26].

We have previously shown that the expression of *Grem1* in the limb bud mesenchyme is controlled by a large genomic landscape downstream of its transcription unit [7]. In fact, several of the classical *limb deformity* mutations that disrupt distal limb bud development and formation of metanephric kidneys in the mouse are caused by deletions affecting this *cis*-regulatory landscape rather than directly altering the *Grem1* gene products [7,13,28]. Molecular and genetic analysis in the mouse identified a 70 kb genomic region located downstream of the *Grem1* transcription unit within the neighbouring *Formin1* (*Fmn1*) gene that is required for *Grem1* expression in the limb bud mesenchyme [7,29]. Detailed analysis of this *Grem1* genomic landscape revealed similarities with the global control region (GCR) that controls the expression of *5’HoxD* genes in the limb bud mesenchyme [6], but did not reveal the structural nature and transacting factors and/or signalling pathways controlling these *cis*-regulatory regions.

To gain further insights into the *Grem1* landscape, the potential *cis*-regulatory activities of the three highest evolutionarily conserved genomic regions within the 70 kb *Grem1* critical region were analysed. In addition to its expression in the posterior mesenchyme, emphasis was also given to the coordinated distal-anterior expansion of *Grem1* expression, which is key to orderly progression of limb bud development [14,30]. Combining BAC with conventional transgenic approaches, we identified a 9 kb genomic region that is able to recapitulate major but not all aspects of the dynamic spatio-temporal *Grem1* expression in the limb bud mesenchyme. This 9 kb region was termed *Grem1 Regulatory Sequence 1* (*GRS1*), and contains a core sequence that is essential to express a *LacZ* transgene under control of a *ß-globin* minimal promoter in a *Grem1*-like pattern. The *GRS1* region drives gene expression into the posterior and subsequently distal-anterior mesenchyme, i.e. reproduces aspects of the distal-anterior expansion of *Grem1* expression during progression of limb bud development. By crossing *GRS1* transgenic mice into different mutant contexts, we establish that the *GRS1* region is controlled by inputs from both the SHH and BMP signalling pathways in limb buds. In addition, chromatin immunoprecipitation (ChIP) shows that GLI3 interacts with the conserved HMCO1 sequences in the *GRS1* region*.* The functionally relevant *cis*-regulatory regions identified by the present and two previous studies [31,32] map to the endogenous DNAse I hypersensitive sites within the *Grem1* genomic landscape recently identified by the ENCODE consortium [33,34]. This indicates that the *Grem1 cis*-regulatory landscape is composed of at least five active *cis*-regulatory regions that control the spatio-temporal expression of *Grem1* in mouse embryos and limb buds.

## Results and Discussion

### Identification of conserved limb bud regulatory regions within the *Grem1* genomic landscape

**Figure 1:**
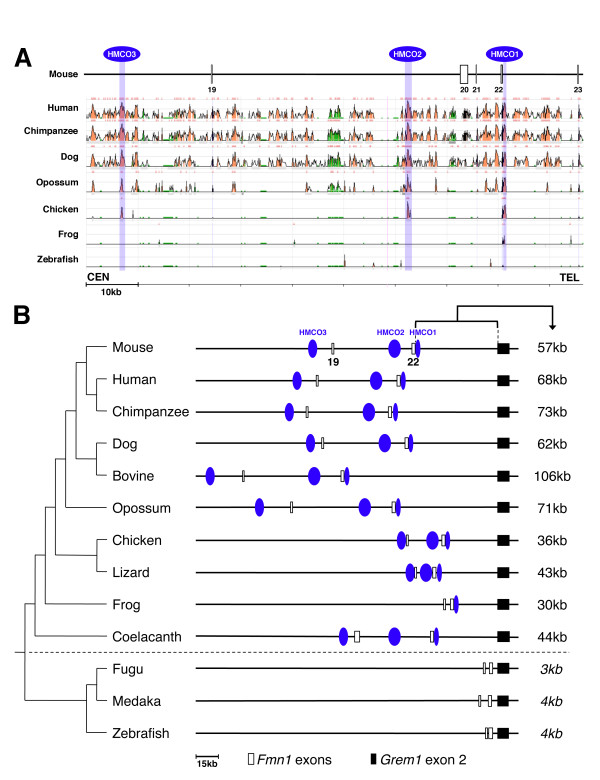
**Identification of three highly conserved non-coding regions in tetrapods and a lobe-finned fish.** (**A**) Sequence alignment of the genomic region critical for *Grem1* expression in mouse limb buds [7] using the ECR browser with the mouse genome release 9 (mm9) as reference genome. The critical genomic region on mouse chromosome 2 is shown in centromeric (CEN) to telomeric (TEL) orientation and is located downstream of the *Grem1* coding exons. As the critical genomic region is part of the *Fmn1* locus, the interspersed *Fmn1* coding exons are indicated as open boxes in the scheme. Three blocks of highly conserved non-coding sequences were identified (HMCO1-3) and are indicated in blue. Conserved coding regions are indicated in black and non-coding regions conserved ≥74% over ≥100 bp are coloured salmon. The peak detected in the region of HMCO1 in the zebrafish genome corresponds to *Fmn1* exon 22. Regions consisting of repetitive sequences are shown in green (see Additional file 2). (**B**) Conserved linkage between the *Grem1* and *Fmn1* loci in vertebrates. Increased intergenic distances correlate with the presence of HMCO regions in tetrapods and coelacanth in contrast to ray-finned fishes. The phylogenetic tree analysis was done with the UCSC multiple alignment functions to align the 3' part of the *Fmn1* locus and the *Grem1* locus from different species. Open boxes represent the orthologous *Fmn1* coding exons 19 and 22, black box represents *Grem1* coding exon 2. The intergenic distances between *Fmn1* orthologous exon 22 and *Grem1* coding exon 2 are indicated to the right of the scheme. ENSEMBL genomes used for alignment: mouse: *M. musculus* (mm10); human: *H. sapiens* (hg19); chimpanzee: *P. troglodytes* (panTro3); dog: *C. familiaris* (canFam2); bovine: *B. taurus* (bosTau6); opossum: *M. domestica* (monDom5); chicken: *G. gallus* (galGal3); lizard: *A. carolinensis* (anoCar2); frog: *X. tropicalis* (xenTro2); coelacanth: *L. chalumnae* (LatCha1); fugu: *T. rubripes* (fr3); medaka: *O. latipes* (oryLat2); zebrafish: *D. rerio* (danRer7).

Using functional genetics in the mouse, we previously identified a ~70 kb region located downstream of *Grem1* that is required for its expression in the limb bud mesenchyme (Figure 1A, [7]). This limb bud *cis*-regulatory region is located between coding exons 19 and 23 of the neighbouring *Fmn1* gene. The Evolutionary Conserved Regions (ECR) genome browser ( http://ecrbrowser.dcode.org/) was used for multiple sequence alignments to compare the mouse genome with different mammalian and the chicken genomes. This analysis revealed several blocks of highly conserved sequences among mammalian species, but only three of them were also highly conserved in the chicken and termed HMCO1, 2 and 3 (Human-Mouse-Chicken-Opossum conserved sequences 1 to 3, Figure 1A). The most conserved parts of these three HMCO core regions are ~80% identical (for details see Additional file 1 and Additional file 2). Of these three regions only HMCO1 is also present in the orthologous genomic region in frogs (Figure 1A), which express *Grem1* during limb bud development [35]. Despite the fact that *Grem1* is expressed during fin bud development [36], no HMCO homologies are present in the zebrafish genome (Figure 1A). ClustalW2 alignments ( http://www.ebi.ac.uk/Tools/msa/clustalw2/) of the *Fmn1Grem1* regions from different species revealed the absence of HMCO homologies in other ray-finned fish species such as fugu and medaka (Figure 1B). In contrast, all three HMCO regions are present in the genome of coelacanth, a lobe-finned fish closely related to tetrapods (Figure 1B and Additional file 1) [37]. As the *Grem1* and *Fmn1* loci are linked in all species analysed (Figure 1B), the distance between the orthologous *Fmn1* coding exon 22 (located adjacent to HMCO1) and *Grem1* coding exon 2 was determined. During evolution, this distance increased as it is smallest in the genomes of ray finned fishes and at least ~10-fold larger in tetrapods and coelacanth (Figure 1B). In addition, the distance between *Fmn1* exon 19 (close to HMCO3) and exon 22 correlates well with the presence or absence of the three HMCO regions (Figure 1B and data not shown). In ray-finned fishes and frogs (lacking all or specifically HMCO2, 3, respectively), the distance is ~3-7 kb, while in mammals and coelacanth it ranges from ~50-70 kb. Thus, the increase of intronic and intergenic regions in the *Fmn1-Grem1* landscape correlates well with appearance of the three HMCO regions during vertebrate evolution. 

**Figure 2:**
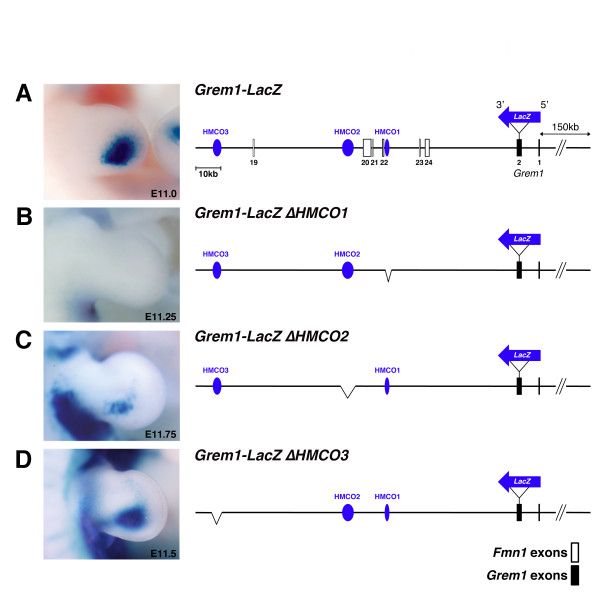
**A BAC-based strategy to assess the requirement of highly conserved non-coding regions (HMCO1-3) for *Grem1* expression in mouse limb buds.** (**A**) A 250 kb BAC spanning the genomic region from *Fmn1* exon 19 to 150 kb up-stream of the *Grem1* transcription is able to drive expression of a *LacZ* transgene (blue arrow; inserted in-frame into the *Grem1* ORF) into the posterior limb bud mesenchyme in transgenic founder embryos. (**B**) Deletion of the HMCO1 core region (520 bp) abolishes the *LacZ* expression. (**C**, **D**) Deletion of the core HMCO2 region (1279 bp) results in reduced *LacZ* expression, while deletion of HMCO3 (924 bp) does not alter the *LacZ* distribution. ß-galactosidase activity colours expressing cells blue.

To determine the requirement of each of these three HMCO regions for *Grem1* expression in mouse limb buds, we used a BAC-based strategy in combination with analysis of transgenic founder embryos (Figure 2A-D and Additional file 3). A 250 kb mouse genomic BAC encoding the critical region and the *Grem1* transcription unit was used to fuse the *LacZ* gene in frame with the *Grem1* ORF [7]. We assessed the expression of the *LacZ* reporter transgene by analysing the spatio-temporal distribution of ß-galactosidase activity (Figure 2A-D). Expression of the *Grem1-LacZ* fusion protein in the forelimb bud mesenchyme was detected in a posterior-distal domain (Figure 2A), mimicking the early endogenous *Grem1* expression rather accurately. Therefore, the *Grem1-LacZ* BAC construct was used to engineer deletions of the three HMCO regions and determine their requirement for *Grem1-LacZ* expression in the posterior mesenchyme. To control for reproducible generation of expressing BAC transgenic founder embryos, the *Grem1-LacZ* BAC (Figure 2A) was injected in parallel to the BACs with engineered deletions (Figure 2B-D). While the control *Grem1-LacZ* BAC was always robustly expressed in the posterior mesenchyme (Figure 2A), deletion of the 520 bp HMCO1 core region resulted in complete loss of *LacZ* expression from limb buds (Figure 2B, n = 5/7 embryos with *LacZ* expression, for details see Additional file 3). In contrast, deletion of the 1279 bp HMCO2 core region only caused partial loss of *LacZ* expression from the posterior limb bud mesenchyme (Figure 2C; Additional file 3). *LacZ* remained expressed normally in the majority of founder embryos carrying a 924 bp deletion of the HMCO3 region (Figure 2 = 3/5 embryos with *LacZ* expression, for details see Additional file 3). Taken together, this BAC transgenic analysis establishes HMCO1 as most critical for *Grem1* expression in the posterior-distal limb bud mesenchyme. As the HMCO1 core region is highly conserved in tetrapods and lobe-finned fish but not ray-finned fish (Figure 1B), it likely represents a *cis*-regulatory region important to tetrapod evolution. The other two HMCO regions might contribute to robust expression of *Grem1LacZ* in the posterior mesenchyme, as in particular the deletion of HMCO2 results in significantly reduced *LacZ* expression (Figure 2C). 

**Figure 3:**
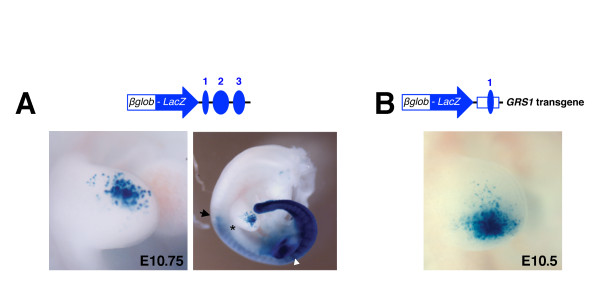
**A 9 kb *GRS1 *transgene encompassing HMCO1 drives *ßglob-LacZ *expression into the posterior limb bud mesenchyme.** (**A**) A transgene encoding the three HMCO core regions downstream of the *ßglob-LacZ* minimal promoter and reporter results in aberrant ß-galactosidase activity in the anterior-distal limb bud mesenchyme. An asterisk marks the posterior border of the forelimb bud. A black arrow marks the anterior border of *LacZ* expression in the trunk. A white arrowhead points to the hindlimb bud. (**B**) The *GRS1*-*ßglob-LacZ* transgene is expressed in the posterior limb bud mesenchyme of transgenic founder embryos. The 9 kb *GRS1* region was inserted downstream of the *ßglob-LacZ* reporter to keep the same arrangement as in the endogenous *Grem1* locus (Figure 2A).

In an attempt to gain further insight into the enhancer potential and possible interactions of HMCO2/3 with HMCO1, conventional transgenic approaches using a minimal human *ß-globin* promoter (*ßglob-LacZ*) were employed. However, neither individual HMCO regions (data not shown) nor in a combination of all three was able to drive robust expression of the *ßglob-LacZ* transgene in the posterior limb bud mesenchyme (Figure 3A, compare to Figure 2A). Expression of the *ßglob-LacZ* transgene under control of all three HMCO core regions resulted in scattered *LacZ* positive cells in the anterior-distal mesenchyme of forelimb buds (left panel, Figure 3A, n = 3/3). In contrast, the transgene was strongly expressed in the posterior embryo including hindlimb buds (right panel, Figure 3A). These results show that a transgene consisting of an array of the HMCO core regions is unable to drive *LacZ* expression into the posterior forelimb bud mesenchyme. This indicates that additional elements of the *Grem1* genomic landscape are required for *Grem1* expression in limb buds.

**Figure 4:**
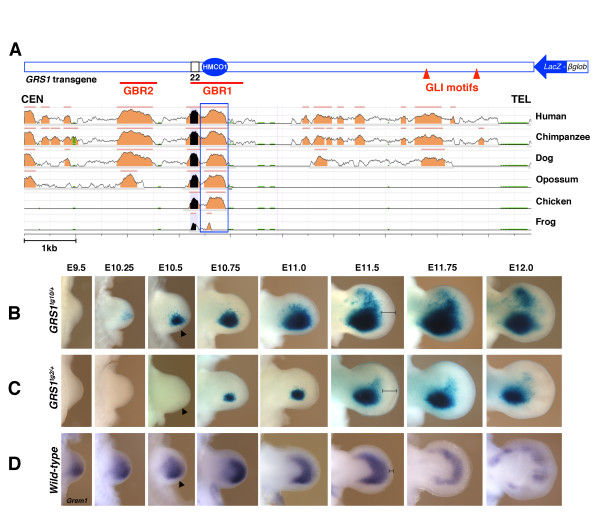
**Comparison of *GRS1 *-dependent ß-galactosidase activity with endogenous *Grem1 *transcripts during limb bud development.** (**A**) ECR browser alignment of the 9 kb *GRS1 region* containing HMCO1. The mouse genome (mm9) is used as reference genome. The conservation threshold was set ≥74% identity over ≥100 bp (see legend to Figure 1A for details). Above the sequence alignment, a schematic representation of the *GRS1-ßglob-LacZ* transgene is shown. GBR: GLI binding region [32]; GLI motifs: consensus GLI binding sites identified by *in silico* analysis. (**BC**) Distribution of ß-galactosidase activity in forelimb buds of heterozygous embryos of the *tg10* (10 copies, *GRS1*^*tg10/+*^, B) and the *tg2* (2 copies, *GRS1*^*tg2/+*^, C) mouse strains between embryonic days E9.5 to E12. (**D**) Distribution of endogenous *Grem1* transcript in the corresponding stages of wild-type forelimb buds. Arrowheads at E10.5 point to the ß-galactosidase activity and *Grem1* transcript in the posterior limb bud mesenchyme. The brackets at E11.5 serve to indicate the distance between the ß-galactosidase activity (**BC**) or *Grem1* expression domain (**D**) and the AER. Note that ß-galactosidase activity fails to expand distally in comparison to the *Grem1* domain.

Next, we assessed to which extent a larger genomic fragment containing HMCO1 and flanking regions could drive *ßglob-LacZ* expression into the posterior limb bud mesenchyme (Figure 3B). Analysis of transgenic founder embryos revealed that this transgenic construct drives robust *ßglob-LacZ* expression in the posterior limb bud mesenchyme (Figure 3B, see also Figure 4), strikingly similar to the domain of the parental *Grem1-LacZ* BAC construct (Figure 2A). Taken together, these results indicate that this *cis*-regulatory region enhances *Grem1* expression in the posterior limb bud mesenchyme, which prompted us to term this 9 kb region *Grem1 Regulatory Sequence 1* (*GRS1*, Figure 4A). *GRS1* encompasses the HMCO1 region next to *Fmn1* coding exon 22 and a further downstream region that is highly conserved in mammals. This region, like HMCO1 overlaps with a previously identified GLI binding region (GBR, [32]). In addition, two potential GLI binding sites were identified by *in silico* analysis (Figure 4A). 

### Spatio-temporal activity of the *GRS1* region during mouse limb bud development

To analyze comparatively the activity of the *GRS1* region with respect to the spatio-temporal regulation of *Grem1* expression, transgenic mouse strains expressing the *GRS1-ßglob-LacZ* reporter construct were established (Figure 4). Seven transgenic founders were obtained. In three independent strains, *LacZ* was expressed in the posterior fore- and hindlimb bud mesenchyme in a pattern comparable to the transgenic founder embryos (Figure 3B and data not shown). Two of these three transgenic strains were analyzed in detail and used to study the spatio-temporal *LacZ* distribution (Figure 4B, C and Additional file 4). Initial analysis revealed that ß-galactosidase activity of the *LacZ* transgene was detected earlier in one strain and is significantly higher than in the other strain (Figure 4B, compare to Figure 4C). As levels did not change during subsequent generations (data not shown), this is likely due to differences in transgene copy number and/or integration site. Therefore, the copy number of both strains was determined by real-time qPCR analysis (Additional file 4). This analysis revealed that ten copies of the *GRS1-ßglob-LacZ* transgene were integrated into the genome of the strain expressing higher *LacZ* levels (*GRS1*^*tg10*^), while only two copies were detected in the *GRS1*^*tg2*^ strain (Additional file 4). In *GRS1*^*tg10/+*^ embryos, ß-galactosidase activity is first detected at ~ E10.25 in the posterior forelimb bud mesenchyme and continuously increases until ~ E11.75 (Figure 4B). From ~ E11.0 onwards, scattered positive cells were detected in the anterior mesenchyme, and this anterior expression increased during distal limb bud outgrowth to form a crescent in the distal-anterior autopod (right panels, Figure 4B). By E11.75, ß-galactosidase activity was rather variable, such that the anterior crescent separated from the posterior domain in some forelimb buds. In *GRS1*^*tg2/+*^ embryos, the spatio-temporal pattern is similar, but expression levels are significantly reduced due to the lower transgene copy number, resulting in detection of ß-galactosidase activity from only ~ E10.5 onwards (Figure 4C, arrowhead). In both strains, *GRS1* drove expression of the *ßglob-LacZ* reporter specifically in the limb bud mesenchyme, despite some low ß-galactosidase activity detected in the developing eyes of *GRS1*^*tg10/+*^ transgenic embryos (Additional file 4). This analysis reveals the robust nature of the *GRS1* region, which functions in positive regulation of *Grem1* expression in the limb bud mesenchyme. The posterior domain of ß-galactosidase activity at E10.5 (Figure 4B) is comparable to the *Grem1* transcript distribution in wild-type limb buds at earlier stages (E9.5-E10.25, Figure 4D). This temporal delay is likely due to postponed transcriptional activation and/or up-regulation of the *GRS1* transgene as the establishment of a posterior *LacZ* expression domain is also only apparent at E10.5 in *GRS1*^*tg10/+*^ transgenic limb buds (Additional file 5). Therefore, additional *cis*-regulatory regions likely control the temporally correct early onset of *Grem1* expression*.* Similar delays in the onset of *LacZ* reporter gene expression have been previously observed by analyzing the ZRS *cis*-regulatory region that controls *Shh* expression in mouse limb buds [38]. During distal progression of limb bud development, endogenous *Grem1* expression expands distal-anterior within the developing handplate and begins to fade by E11.75 (Figure 4D), due to termination of the SHH/GREM1/AER-FGF feedback loop and GLI3-mediated repression in the anterior limb bud mesenchyme [11,26,39,40]. In contrast, ß-galactosidase activity remains high in the posterior limb bud mesenchyme of *GRS1* transgenic embryos and the distal-anterior expansion of its expression is significantly delayed and only occurs as transcription of the endogenous *Grem1* locus is starting to terminate (E11.5 onwards Figure 4B, C, compare to Figure 4D).

**Figure 5:**
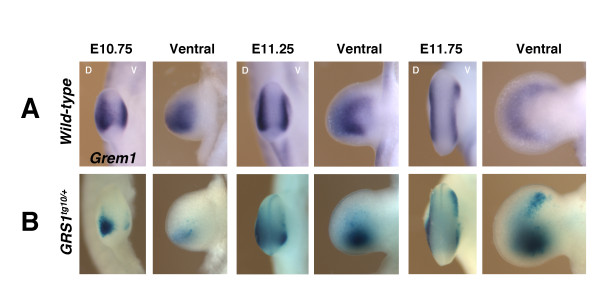
**Dorsal and ventral restriction of *GRS1*-mediated ß-galactosidase activity.** (**A**) *Grem1* transcript remains restricted to the dorsal and ventral forelimb bud mesenchyme in wild-type embryos throughout limb bud development. (**B**) *GRS1*-mediated expression of *ßglob-LacZ* is able to correctly restrict ß-galactosidase activity along the dorso-ventral limb bud axis. D: dorsal; V: ventral.

*Grem1* expression is restricted to dorsal and ventral limb bud mesenchyme and excluded from the chondrogenic core mesenchyme (Figure 5A), which is relevant to BMP-mediated induction of chondrogenesis (ref. [26,41] and Benazet et al., submitted). Furthermore, the LIM-homeodomain transcription factors Lhx2 and Lhx9 have been implicated in regulating *Grem1* expression predominantly in the ventral limb bud mesenchyme in response to SHH signalling [42]. Therefore, the extent to which the dorso-ventral transcript distribution is maintained by the *GRS1* transgene was assessed (Figure 5B). Indeed, the expression of both the high (Figure 5B) and low copy transgenes (Additional file 6) remained excluded from the core mesenchyme throughout limb bud outgrowth and patterning. Similar to *Grem1* transcripts, ß-galactosidase activity is higher dorsally than ventrally (Figure 5B, compare to Figure 5A). By E11.75, ß-galactosidase activity expands anteriorly in both the dorsal and ventral mesenchyme (E11.75, Figure 5B), reminiscent of the crescent of *Grem1* transcript (Figure 5A and see before), which indicates that the *GRS1* contains *cis*-regulatory regions controlled by Lhx transcription factors [42]. Taken together, the results shown in Figures 4 and 5 establish that the 9 kb *GRS1 cis*-regulatory region is able to recapitulate major aspects of *Grem1* expression in the limb bud mesenchyme. In particular, the *GRS1* also recapitulates aspects of the distal-anterior expansion of *Grem1* expression in limb buds (Figure 4,5), which was not observed in previous attempts to identify *cis*-regulatory regions that control *Grem1* expression in limb buds [7,32]. The fact that the *GRS1* transgene recapitulates some aspects of this distal-anterior expansion is important, as *Grem1* transcription normally expands anteriorly in register with posterior AER-FGFs and allows propagation of SHH/GREM1/AER-FGF signalling [14,30]. However, the delay in activation and lack of termination of the *GRS1* transgene (Figure 4) indicates that other regulatory inputs from the *Grem1* genomic landscape are required to regulate its dynamic expression in mouse limb buds. 

### The *GRS1 cis*-regulatory region integrates inputs from the SHH and BMP signalling pathways

**Figure 6:**
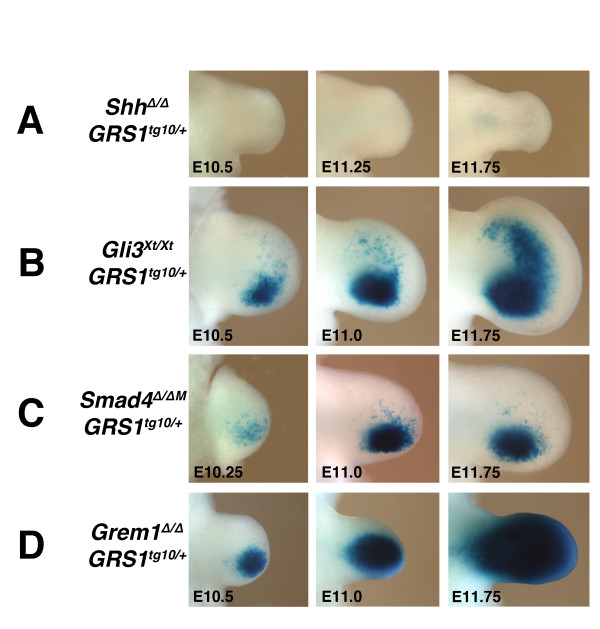
**The *GRS1 *transgene is controlled by both SHH and BMP activity.** (**A**) *GRS1*-dependent ß-galactosidase activity is completely lost from *Shh*-deficient limb buds (*Shh*^*Δ/Δ*^*GRS1*^*tg10/+*^). (**B**) In contrast, inactivation of *Gli3* results in anterior up-regulation of ß-galactosidase activity at E11.75 (*Gli3*^*Xt/Xt*^*GRS1*^*tg10/+*^). (**C**) ß-galactosidase activity fails to expand anteriorly in *Smad4*-deficient limb buds (*Smad4*^*Δ/ΔM*^*GRS1*^*tg10/+*^). (**D**) ß-galactosidase activity is up-regulated in *Grem1*-deficient limb buds from E11.0 onward (*Grem1*^*Δ/Δ*^*GRS1*^*tg10/+*^).

During limb bud initiation, mesenchymal BMP4 signal transduction is likely required to activate *Grem1* expression in the posterior mesenchyme, while SHH is primarily required for up-regulation and distal-anterior expansion during progression of limb bud development [11,14,43]. Furthermore, GLI3 in the anterior mesenchyme and AER-FGF signal transduction are required to restrict and eventually terminate *Grem1* expression (starting ~ E11.5-11.75) [26,40]. Therefore, *GRS1*^*tg10/+*^ embryos lacking key components of both the SHH and BMP signalling pathway in their limb buds were generated to gain insight into the possible direct impact of these main signalling pathways on the *GRS1* element (Figure 6). Analysis of *Shh*^*Δ/Δ*^*GRS1*^*tg10/+*^ embryos revealed the complete absence of ß-galactosidase activity in *Shh*-deficient forelimb buds (Figure 6A). This contrasts with the endogenous *Grem1* expression*,* which is activated but not maintained in *Shh*-deficient limb buds [11,14]. These results show that activation of the *GRS1* region depends on SHH signalling, which indicated that it could also participate in ectopic *Grem1* activation due to anterior ectopic SHH signalling in mouse [14] and chicken limb buds [12]. Furthermore, GLI3 proteins interact with specific *cis*-regulatory regions in the *Grem1* genomic landscape [32] and genetic analysis has shown that *Gli3* is essential for the spatio-temporally controlled restriction and subsequent termination of *Grem1* expression in the distal anterior mesenchyme [25,26,44]. In *Gli3*-deficient *GRS1* transgenic (*Gli3*^*Xt/Xt*^*GRS1*^*tg10/+*^) forelimb buds, ß-galactosidase activity in the posterior limb buds is comparable to *GRS1*^*tg10/+*^ limb buds, while expression in the anterior mesenchyme is significantly increased by E11.75 (Figure 6B, compare to right-most panels, Figure 4B). This late up-regulation of anterior ß-galactosidase activity indicates that *GRS1* is required for GLI3-mediated termination of *Grem1* expression in the anterior limb bud mesenchyme, which is essential for the spatio-temporally correct initiation of mesenchymal condensations and chondrogenic differentiation of anterior digits [26]. 

BMPs control *Grem1* activation in the posterior limb bud mesenchyme and directly modulate its expression as part of the self-regulatory SHH/GREM1/AER-FGF feedback signalling system. Therefore, a conditional *Smad4* loss-of-function allele [45] in combination with the *Prx1*-Cre recombinase strain [46] was used to inactivate *Smad4* (*Smad4*^*Δ/ΔM*^) and thereby canonical BMP signal transduction in the limb bud mesenchyme. In *Smad4*^*Δ/ΔM*^ mutant limb buds, endogenous *Grem1* expression is down-regulated but not lost, while *Shh* expression remains (Benazet et al., submitted). In *Smad4*^*Δ/ΔM*^*GRS1*^*tg10/+*^ forelimb buds, ß-galactosidase activity appears normal in the posterior mesenchyme, while distal-anterior expansion fails to occur (Figure 6C, compare to Figure 4B). This indicates that *GRS1* activity is modulated by SMAD4-mediated signal transduction during the progression of limb bud development. These results indicate that also the distal-anterior expansion of *Grem1* expression depends on BMP activity and agree with the observation that genetic lowering of *Bmp4* results in a global reduction of *Grem1* expression in the limb bud mesenchyme [11].

Finally, in *Grem1-*deficient embryos, BMP activity is increased due to reduced BMP antagonism, which results in up-regulation of non-functional *Grem1* transcript [13]. As BMP signal transduction modulates *GRS1* activity during advanced limb bud development (Figure 6C), we also determined the potential influence of *Grem1* deficiency on *GRS1* activity. In *Grem1*^*Δ/Δ*^*GRS1*^*tg10/+*^ E10.5 limb buds ß-galactosidase activity is initially similar to *GRS1*^*tg10/+*^ limb buds (Figure 6D, compare to Figure 4B). Subsequently, ß-galactosidase activity is increased and by E11.75 the entire *Grem1*^*Δ/Δ*^*GRS1*^*tg10/+*^ limb bud is positive (Figure 6D). These alterations of ß-galactosidase activity in *Smad4*^*Δ/ΔM*^ and *Grem1*^*Δ/Δ*^ forelimb buds reveal that *GRS1* activity is extensively modulated by changes in BMP activity, in particular also the anterior expansion of its expression. Taken together, this genetic analysis (Figure 6) shows that *GRS1* activity critically depends on SHH and is modulated extensively by BMP signal transduction. This analysis identifies the *GRS1*, located ~70 kb downstream of the *Grem1* transcription unit as a *cis*-regulatory region that integrates inputs by both SHH and BMP signal transduction. However, as previous analysis provided good evidence that *Grem1* is activated by mesenchymal BMP signalling upstream of establishing the SHH/FGF feedback loop [11], these studies point to the existence of additional unknown BMP response regions that activate *Grem1* expression in the posterior limb bud mesenchyme.

### Endogenous GLI3 proteins are part of the chromatin complexes interacting with specific parts of the *GRS1* region in mouse limb buds

**Figure 7:**
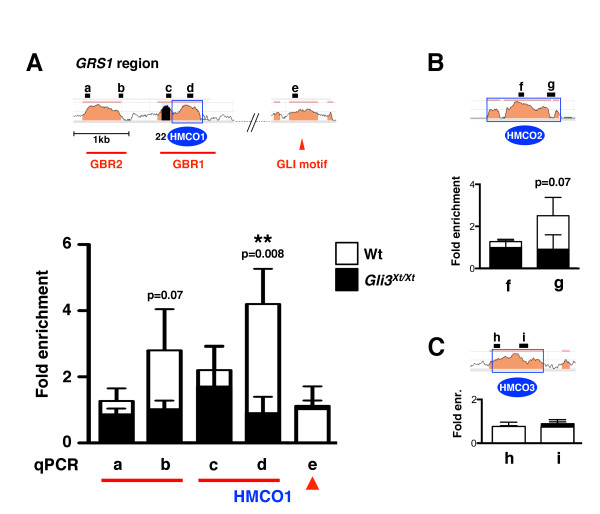
**Endogenous GLI3 complexes bind to specific HMCO and GBR regions in the *Grem1 *genomic landscape.** (**A**) Several amplicons were designed to detect the conserved and functionally relevant regions within the *GRS1* region. The exact genomic locations of all amplicons are listed in Additional file 7. The potential interaction of endogenous GLI3 proteins with these critical amplicons was analysed by ChIP-qPCR analysis of wild-type (open bars) and *Gli3*^*Xt/Xt*^ (black) limb buds at E11.5. Two stars indicate significant enrichment of amplicon “d” in the HMCO1 region (p = 0.008). (**B**) GLI3 ChIP-qPCR analysis of the HMCO2 region. Amplicons “f” and “g” are located inside HMCO2 and the previously identified GBR3 [32]. (**C**) GLI3 ChIP-qPCR analysis of HMCO3. All values are shown as mean ± SD.

The observed loss of *GRS1* activity in *Shh-*deficient limb buds (Figure 6A) indicated that the *GRS1* region could be regulated directly by GLI proteins. Indeed, two GLI binding regions without consensus GLI binding motif (GBR1 and GBR2, [32]) were previously mapped to the 9 kb *GRS1* region using transgene-mediated expression of an epitope-tagged GLI3 transgene in combination with chromatin immunoprecipitation (ChIP). To study the potential interaction of endogenous GLI3 proteins with the *GRS1* region in its normal genomic context in wild-type limb buds, ChIP analyses using antibodies that detect both the GLI3 activator and repressor protein isoforms were performed [26,47]. GLI3 ChIP using extracts of wild-type and *Gli3*-deficient limb buds at E11.5 revealed significant enrichment of one specific region within *GRS1* by real-time qPCR analysis (Figure 7A). A significant ~4-fold enrichment of an amplicon located within HMCO1 was observed by comparing wild-type to *Gli3*-deficient limb buds (amplicon “d”; p = 0.008) and to a less conserved region (amplicon “e”; located outside HMCO1 close to a putative GLI binding site; Figure 7A). In contrast, no significant enrichments of the other amplicons located in *GRS1* were detected (Figure 7A). As the amplicons located in HMCO2 (Figure 7B) and HMCO3 (Figure 7C) were also not significantly enriched, the GLI3-containing chromatin complexes appear to interact rather specifically with a region within the highly conserved HMCO1 in wild-type limb buds. These results not only corroborates the identity of previously identified GBR1 [32], but indicate that this region mediates the effects of SHH signal transduction on *GRS1* activity in the posterior limb bud mesenchyme. 

### Spatio-temporal *Grem1* expression is regulated by the interaction of multiple *cis*-regulatory regions far downstream of the transcription unit

Previous genetic analysis showed that *Grem1* transcription in limb buds is initiated by BMP signalling and up-regulated under the influence of SHH and AER-FGF signaling as limb bud development progresses [11]. Finally, *Grem1* expression is terminated concomitantly with the initiation of digit formation by high levels of FGF signal transduction and GLI3-mediated repression in the anterior mouse limb bud mesenchyme [26,40]. This dynamic spatio-temporal regulation of *Grem1* expression indicates that the activity of different *cis*-regulatory elements may change over time. The spatio-temporal activity of the *GRS1* transgene (Figure 4B) indicates that it is primarily regulated by the SHH/GREM1/AER-FGF feedback signaling system as limb bud development progresses. In particular, its activity is first apparent when the SHH/GREM1/AER-FGF feedback is already established, and the expected termination does not occur, as ß-galactosidase activity remains in the posterior mesenchyme after the endogenous *Grem1* transcripts have been down-regulated (Figure 4B, D). These results indicate that FGF-mediated termination of *Grem1* expression and the underlying self-regulatory feedback signalling system [40] does not occur by FGF signal transduction impacting the *GRS1* region. In addition, neither the 70 kb critical region of the *Grem1* landscape (*Grem1-LacZ*, Figure 2A) nor transgenes derived from this region (Figures. 2,3,4 and ref. [32]) are able to accurately recapitulate the entire spatio-temporal distribution of *Grem1* transcript in the limb bud mesenchyme. Therefore, either the interactions among these elements or additional as yet unknown *cis*-regulatory regions located outside the critical region must also participate in the regulation of *Grem1* expression. Indeed, there is circumstantial evidence for the latter, as deletion of the GC-rich *Fmn1* coding exon 9 (located ~200 kb downstream of the *Grem1* transcription unit) results in non-complementation with a *Grem1* null allele, causing a hypomorphic *Grem1* limb skeletal phenotype, and significantly reduced *Grem1* expression (ref. [29] and A.Z and R.Z., unpublished data).

**Figure 8:**
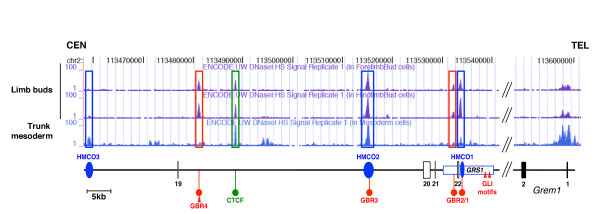
**The *Grem1 *genomic landscape harbours multiple distant *cis*-regulatory regions that orchestrate *Grem1 *transcription in limb buds.** Mapping of the DNAse I hypersensitive (HS) sites in fore-, hindlimb bud and trunk mesoderm of mouse embryos at E11.5 (ENCODE Group, University of Washington, [33,34]). A schematic representation of the *cis*-regulatory landscape that regulates *Grem1* expression in the limb bud mesenchyme is shown below the HS site tracks. Interestingly, the major HS sites co-localise with regions that are functionally required, highly conserved (HMCO1-3, blue), and/or overlap with experimentally defined GBRs (red, ref. [32] and this study) or a CTCF binding region (green, [31]). Red arrowheads indicate consensus GLI binding sites identified by *in silico* analysis. HMCO1 and HMCO2 overlap with limb bud specific HS sites, while HMCO3 overlaps with a trunk mesodermal HS site. For exact coordinates, see Additional file 8.

Mapping of DNAse I hypersensitive (HS) sites is used to identify active regulatory regions in chromatin (reviewed in ref. [48]). Recently, the ENCODE consortium has done genome-wide mapping of HS sites in several mouse tissues including limb buds (ENCODE Group, University of Washington) [33,34]. Interestingly, this analysis revealed three HS sites within the GBRs of the *GRS1* and HMCO2, while no HS sites mapping to HMCO3 were detected in limb buds at E11.5 (Figure 8). The five HS sites mapped by the ENCODE consortium in the *Grem1* genomic landscape overlap with the *cis*-regulatory elements required for *Grem1* expression in mouse limb buds (Figure 8). The HS site mapping to HMCO1 overlaps well with the amplicon enriched by GLI3-ChIP analysis (Figure 7A). In fact, all HS sites in the *Grem1* genomic landscape overlap either with the GBR regions interacting with GLI3 [26,32] or CTCF binding sites [31]. Analysis of *Ctcf*-deficient limb buds revealed its requirement for up-regulation and distal-anterior expansion of *Grem1* expression [31]. Interestingly, the HS site overlapping with GBR4 [32] is not conserved between mammals and birds (Figure 8 and data not shown). While the early GBR4 activity is comparable to *GRS1* in the posterior mesenchyme (Figure 4B), it does not support distal-anterior expansion of the expression during subsequent development [32]. Thus, the interaction of the GBR4 and *GRS1 cis*-regulatory regions could provide *Grem1* expression with the necessary robustness and evolutionary plasticity, as has been postulated for the transcriptional regulation of *HoxD* genes by multiple interacting regulatory regions during limb bud development [8]. Taken together, these studies establish that the five HS sites in the *Grem1* genomic landscape are functionally relevant for *Grem1* expression in mouse limb buds, but not all of them are evolutionary highly conserved. Indeed, experimental and comparative evolutionary evidence indicates that alterations in the spatio-temporal expression of *Grem1,* and thereby the activity of the SHH/GREM1/AER-FGF feedback loop, likely contributes significantly to variations in digit numbers and morphologies (reviewed in ref. [49]). 

## Conclusions

In this study, we identify *GRS1* as a distant *cis*-regulatory region that encompasses the highly conserved HMCO1 region and show that it controls important aspects of *Grem1* expression in the posterior and distal-anterior limb bud mesenchyme. *GRS1* activity depends critically on SHH signalling and endogenous GLI3 protein complexes interact with specific regions within HMCO1 in wild-type limb buds. In addition, the anterior expansion of *GRS1* activity is also regulated by BMP signal transduction. These results together with previous studies reveal the large genomic architecture composed of several distant *cis*-regulatory regions that control the highly dynamic *Grem1* expression in response to signalling inputs from the BMP, SHH and FGF pathways. It is likely that the interactions among these dispersed *cis*-regulatory regions provide the dynamic *Grem1* expression in limb buds with the necessary robustness, but at the same time allow for evolutionary plasticity of its expression [8].

## Methods

### Ethics Statement

All the experiments were conducted in strict accordance with Swiss law following the 3R principles and the conduct defined by the Basel Declaration. All studies involving mice were classified as grade zero by the Animal Welfare and Ethics Commission of both cantons of Basel and Argovia, which implies minimal suffering.

### Mouse strains

The *Shh* loss-of-function allele [50], the *Gli3 extra-toes-*J allele [51], the *Grem1* null allele [13] and the *GRS1*^*tg/+*^ alleles (this study) were maintained in an NMRI background, while the *Smad4* conditional allele (*Smad4*^*flox*^, [45]) and the *Prx1-*Cre transgene [46] were maintained in a C57BL/6 J background.

### BAC modifications

BAC constructs were engineered using ET cloning as previously described [7]. The deletions ΔHMCO1, ΔHMCO2 and ΔHMCO3 were induced in the *Grem1-LacZ* BAC clone using a *Kanamycin* resistance selection cassette*.* All primer sequences are available upon request. See Additional file 8 for the genomic coordinates of HMCO1-3.

### *ßglob-LacZ* transgene constructs

An expression cassette was constructed in pKS-Bluescript using *LacZeocin* reporter under control of the minimal human *ß*-globin promoter sequences [52]. Various combinations of HMCO sequences were inserted downstream into this cassette. The HMCO1, -2 and -3 core sequences were amplified by PCR directly from mouse genomic DNA (primers available on request). The 9 kb *GRS1* region was initially subcloned as a 9.6 kb *Bgl2* fragment from BAC RP23-113 H17 (chr2: 113,611,499-113,847,299 (mm10). BacPac Resources, Children’s Hospital, Oakland, USA). All constructs were linearized with *Ksp1* before microinjection.

### Generation of transgenic founder embryos and transgenic strains

BAC and *ßglob-LacZ* transgenic constructs were injected into the pronucleus of fertilized mouse eggs. Several founder embryos for each construct were scored for ß-galactosidase activity (Additional file 3). For the *GRS1ßglob-LacZ* transgene, seven independent transgenic mouse strains were established by crossing founders into the NMRI background. Transgene copy numbers were determined by real time qPCR [53] using the Bio-Rad CFX96 Real-Time PCR System in combination with the iQ SYBR Green Supermix (Bio-Rad). For each mouse, 20 ng of genomic DNA were analysed in triplicate. The primers to amplify the *DBH* genomic region were used for normalization and non-transgenic littermate DNA was used as control. The normalized control levels were set to 2 as the primers also amplified both alleles of the endogenous HMCO1 regions. The 2^-ΔΔCt^ formula was used to calculate and normalize transgene copy numbers. In addition to three different F1 males, three embryos from the F2 and three embryos from the F3 generation were analysed for the two transgenic strains shown to ascertain stable transmission of the transgenes. Primers sequences are available on request.

### Detection of ß-galactosidase activity

Embryos were isolated in ice-cold PBS and staged according to somites numbers, then fixed in 1% formaldehyde, 0.2% glutaraldehyde, 0.02% NP40, 0.01% sodium deoxycholate in 1x PBS for 20–30 minutes at 4°C. Subsequently, embryos were washed three times in 1x PBS for 5 minutes at room temperature and incubated overnight at 37°C and in the dark in 1 mg/mL X-Gal, 0.25 mM K3Fe(CN6), 0.25 mM K4Fe(CN6), 0.01% NP-40, 0.4 mM MgCl2 and 1% sodium deoxycholate to detect ß-galactosidase activity, which colours cells blue. To stop the reaction, embryos were washed three times in 1x PBS for 5 minutes each at room temperature.

### Chromatin Immunoprecipitation (ChIP)

Forelimbs and hindlimbs of 10 wild-type or *Gli3*^*Xt/Xt*^ embryos at E11.5 were dissected and processed for ChIP as described [26] using a polyclonal anti-GLI3 antibody (#2676, Genentech, [47]). To compute the level of enrichment of a given region, the Ct values of both input and ChIP samples were compared with those of a negative control amplicon located in the mouse *ß-actin* locus [21]. All results (mean ± SD) were obtained by analysing three completely independent experiments per genotype. The significance of all differences was assessed using the two-tailed, non-parametric Mann–Whitney test. The coordinates of the relevant amplification are shown in Additional file 7.

All genomic sequence alignements of the *Fmn1-Grem1* locus region were performed using the ECR browser [54] and the ClustalW2 program [55].

## Authors’ contributions

AZ conceived most of the experiments and wrote the manuscript together with RZ. AZ also carried out most of the genetic and transgenic analysis together with FL. FL also prepared the figures for the manuscript. FL, JLR and NM performed the *in silico* analysis. JLR carried out the ChIP analysis to detect the endogenous GLI3 protein complexes. NM contributed in the initial phase of this project (generation of transgenic constructs and initial analysis of founder embryos). CK generated the transgenic founders for all constructs. RZ conceived part of the study and wrote the manuscript together with AZ. All authors read and approved the final manuscript.

## Supplementary Material

Additional file 1 **Figure S1.** Conservation of the HMCO core regions. ClustalW2 multiple sequence alignment of the HMCO1, HMCO2 and HMCO3 core regions of mouse (mm10), human (hg19), chimpanzee (panTro3), dog (canFam2), bovine (bosTau6), opossum (monDom5), chicken (galGal3), lizard (anoCar2), frog (xenTro2), and coelacanth (LatCha1) genomes The corresponding genomic coordinates are indicated. HMCO1: 73 of 149 nucleotides were conserved in all species; HMCO2: 182/298 conserved nucleotides, HMCO3: 45/137 conserved nucleotides. Click here for file

Additional file 2 **Table S1.** Genomic coordinates for the sequence comparisons shown in Figure 1A. Click here for file

Additional file 3 **Table S2.** Analysis of transgenic founder embryo. Click here for file

Additional file 4 **Figure S2.** Limb bud mesenchymal expression of *GRS1-ßglob-LacZ* transgene in two independent transgenic mouse strains. (A) Expression of the *GRS1-ßglob-LacZ* transgene is restricted to limb buds, with expression initiating earlier in forelimb than hindlimb buds. Note the differences in ß-galactosidase activity in *GRS1*^*tg10/+*^ (left panel) and *GRS1*^*tg2/+*^ (right panel) transgenic mouse embryos. (B) Using real-time qPCR, the transgene copy numbers in both the *GRS1*^*tg10/+*^ and *GRS1*^*tg2/+*^ mouse strains were determined in comparison to wild-type mice (carrying 2 copies of the endogenous *GRS1* regions). This analysis revealed that the *GRS1*^*tg10/+*^ strain carries 10 copies and *GRS1*^*tg2/+*^ 2 copies of the transgene, respectively. Click here for file

Additional file 5 **Figure S3.** Comparison of the *LacZ* mRNA and ß-galactosidase reporter activity in early limb buds of *GRS1*^*tg10/+*^ embryos. Distribution of ß-galactosidase activity (A) and *LacZ* transcripts (B) in forelimb buds of *GRS1*^*tg10/+*^ embryos at E10.0 and E10.5. Arrowheads point to the posterior expression domains. Click here for file

Additional file 6 **Figure S4.** Dorso-ventral distribution of ß-galactosidase activity in forelimb buds expressing the *GRS1*^*tg2/+*^ transgene. The *GRS1*^*tg2/+*^ transgene respects the dorsal and ventral restriction of the mesenchymal expression domains. Note that overall expression is significantly lower than in *GRS1*^*tg10/+*^ limb buds (compare to Figure 5B). D: dorsal, V: ventral. Click here for file

Additional file 7 **Table S3.** qPCR amplicons for GLI3 ChIP analysis. Click here for file

Additional file 8 **Table S4.** Genomic coordinates of identified Grem1 regulatory regions. Click here for file
