# Supplementary material for: Conserved cis-regulatory regions in a large genomic landscape control SHH and BMP-regulated Gremlin1 expression in mouse limb buds
Source: BMC Dev Biol. 2012 Aug 13;12:23. doi: 10.1186/1471-213X-12-23 (PMC3541112; doi:10.1186/1471-213X-12-23)
Supplement: Additional file 1 — Figure S1. Conservation of the HMCO core regions. ClustalW2 multiple sequence alignment of the HMCO1, HMCO2 and HMCO3 core regions of mouse (mm10), human (hg19), chimpanzee (panTro3), dog (canFam2), bovine (bosTau6), opossum (monDom5), chicken (galGal3), lizard (anoCar2), frog (xenTro2), and coelacanth (LatCha1) genomes The corresponding genomic coordinates are indicated. HMCO1: 73 of 149 nucleotides were conserved in all species; HMCO2: 182/298 conserved nucleotides, HMCO3: 45/137 conserved nucleotides. [file 1471-213X-12-23-S1.pdf]

Fig. S1

HMCO1 conserved core

```
Mouse           -------TGCAGCCCTTA---TGGGCAAGATACATTCACATGATCTGATAAAGCTCCA-T    2:113693360
Human           -------TGCTGTCCACA----CAGGCAGATATGCTCACATGATCTGATAA-GTTACA-C    15:33090823
Chimpanzee      -------TGCTGTCCACA----CAGGCAGATATGCTCACATGATCTGATAA-GTTACA-C    15:29568630
Dog             GGAGGTGTGCTATCCACA----CTAGCAGATA-GCTCCCGTGGTCTGCTAA-GTTACA-T    30:5150165
Bovine          -------TGCTGTCCACA----CAGGCAGATACTCTCACGTGATCTGATAA-GTTACA-C    10:29933362
Opossum         -------TGCTGCCCCCACCTCAAGCAGATAAACACACATGATCTGATAA-GTTACA-C    1:188935233
Chicken         -------TGCTGTCCCCA----CAAAAAGATAAACACATATAATCTGATAA-ATTACA-C    5:32849615
Lizard          -------TGCTGTCCCTG----CAAAAAGATAAACACGTATAATCTGATAA-ATTACA-C    1:32921655
Frog            --------------------------AAGATAAACACGCCAGATCTGATAA-GTTACACT    GL172673.1:3687453
Coelacanth      -------TGCTGTCCTCT----CAAAA-GATAAATACACATAATCTGATAA-GTTACA-C    JH126706.1:163591
                                            ****    *      **** ***   * **

Mouse           TCTCTTAGCCCCTTCCAATTACTAGCTAGTAAA-TGAAAGGCGAGTTGTGCTAATTTGCA    2:113693419
Human           TTTCTTAACCCCTTCCAATTACTAGCTAGTAAA-TGAAAGGAGGGTTGTGCTAATTTGCA    15:33090750
Chimpanzee      TTTCTTAACCCCTTCCAATTACTAGCTAGTAAA-TGAAAGGAGGGTTGTGCTAATTTGCA    15:29568557
Dog             GTTCTTCACCCCTTCCAATTACAAGCCAGTAAA-TGAAAGGGGAGCAGCGCTAATTTGCA    30:5150224
Bovine          TTTCTTAACCCCTTCCAATTACTAGCTAGTAAA-TGAAAGGGGAGCTGTGCTAATTTGCA    10:29933421
Opossum         TTTCTTAACCCCTTCCAATTACTAGCTAGTAAAATGAAAGGGGAGCAGTGCTAATTTGCA    1:188935293
Chicken         TTTTGTAACCCCTTCCAATTACTAGCTAGTAAAATGAAAGGGGAGCTGTGCTAATTTGTA    5:32849675
Lizard          TTTCTTAACCCCTTCCAATTACTAGCTAGTAAAATGAAAGGGGAGCTGTACTAATTTGTA    1:32921606
Frog            TTTCTTTACCACTCCCAATTACTTGCTAGTAAAATGAAAGGAGACCTCAGCTAATTTGGA    GL172673.1:3687391
Coelacanth      TTTCTTAACCCCTTCCAATTACTAGCTAGTAAAGTGAAAGGGGAGCTGTGCTAATTTGTA    JH126706.1:163651
                  *  *  ** ** ********  ** ****** ******* *       ******** *

Mouse           ACAGATTGCTTCTC-TGTGTATTTTCACATCGGCCCAAGCTAAATGAGGCAGCCTTGCTG    2:113693478
Human           ACAGATTGCTTCTC-TGTGTATTTTCATGTTGGCCCAGGCTAAATGAGGCAGCCTTGCTG    15:33090707
Chimpanzee      ACAGATTGCTTCTC-TGTGTATTTTCATGTTGGCCCAGGCTAAATGAGGCAGCCTTGCTG    15:29568501
Dog             ACAGATTGCTTCAC-TGTGTATTTTCACTTGGGCCGAGGCTAAATGAGGCCGCCTTAGTG    30:5150283
Bovine          ACAGATTGCTTCTC-TGTGTATTTTCATGTCGGCCCAAGCTAAATGAGGCAGCCTTGCTG    10:29933480
Opossum         ACAGATTGCTTCTC-TGGGCATTCTCATGTTGGGCCAGCCTAAATGAGGTGGTATTGCTG    1:188935352
Chicken         ACAGATTGCTTCTC-TGTGCATTTTCATGTTGAACTAGCCTAAATGAGGCGATATTGCTG    5:32849734
Lizard          ACAGATTGCTTCTC-TTTGCATTTTCATGTAGGACTGCCCTAAATGAGGCAAGATTGCTG    1:32921536
Frog            CCAGATTTTTT-----------TTTCTCTTTGG-------TGTCTGAG------TTAC--    GL172673.1:3687349
Coelacanth      ACAGATTGCTTCTCCTGTGCATTTTCATGTTGAACTAGCCCAAATGAGGTGATATTACTG    JH126706.1:163711
                 ******  **           * **   * *            ****      **
```

HMCO2 conserved core

```
Mouse           --------------------------------------------TGGCAAAGAAACAGAT    2:113674550
Human           --------------------------------------------TAGCAAAGAAACAGAT    15:33111832
Chimpanzee      --------------------------------------------TAGCAAAGAAACAGAT    15:29589602
Dog             --------------------------------------------TAGCAAAGAAACAGAT    30:5129417
Bovine          --------------------------------------------AAGCAAAGAAACAGAT    10:29908339
Opossum         --------------------------------------------TAGCAAAGAAACAGAT    1:188909582
Chicken         --------------------------------------------TAGCAAAGAAACAGAT    5:32836402
Lizard          --------------------------------------------GTGCAAAGAAACAGAT    1:32929136
Coelacanth      TGCTTCTTAAAAAAATTCACTGCTTTTTATAATAAAAAGTAAGTAACAAAGAAACAGAT    JH126706.1:131370
                                                               *************

Mouse           GTGTCCCACATAAAACATGCAATAAATCTTGTCCCA-CAAATTCTTTGCTCTGAACTTTC    2:113674609
Human           GTGTCCCATATAAAATATGCAATAAATCTTGTCCCA-CAAATTCTTTGCTCTGAACTTTC    15:33111765
Chimpanzee      GTGTCCCATATAAAATATGCAATAAATCTTGTCCCA-CAAATTCTTTGCTCTGAACTTTC    15:29589535
Dog             GTATCCCCTATAAAATATGCAATAAATCTTGTCCCA-CAGATTCTTTGCTCTGAACTTTC    30:5129476
Bovine          GTGTCCCATATAAAATATGCAATAAATCTTGTCCCA-CAAATTCTTTGCTCTGAACTTTC    10:29908398
Opossum         GTGTCCCATATAAAATATGCAATAAATCTTGTCCCAACAAATTCTTTGCTCTGGACTTTC    1:188909642
Chicken         GTGTCCCATATAAAATATGCCATAAATCTTGTCTCA-CAAATTCTTTTCTCTGAACTTTC    5:32836461
Lizard          GTGCTCCATATAAAATACACTATAAATCTTATCTTG-CAATTTCTTTGCTCCAAACTCTC    1:32929077
Coelacanth      GTGTACCATATAAAATATGCAATAAATCTTGTCCCA-CAAATTCTTTGCCCTGGACTCTC    JH126706.1:131429
                **   **  ****** *  * ********* **    **  ****** * *   *** **

Mouse           TGACAGAAACTGTCAAATATCCA-CTTTAATTCAGGTATAAAATGTTCACTCCGTGTCAT    2:113674668
Human           TGACAGAAACTGTCAAATATCCA-CTTTAATTCAGGTATAAAATGTTCACTCCATGTCAT    15:33111709
Chimpanzee      TGACAGAAACTGTCAAATATCCA-CTTTAATTCAGGTATAAAATGTTCACTCCATGTCAT    15:29589476
Dog             TGACAGAAACTGTCAAATATCCA-CTTTAATTCAGGTATAAACTGTTCACTCCGTGTCAT    30:5129535
Bovine          TGACAGAAACTGTCAAATATCCA-CTTTAATTCAGGTATAAAATGTTCACTCCGTGTCAT    10:299083457
Opossum         TGACAGAAACTGTCAAATATCCA-CTTTAATTCAGGTATAAAATGTTCACTCTGTTTCAT    1:188909701
Chicken         TGACAGAAACTGTCAAATATCCA-CTTTAATTCAAGTATAAAATGTTCACTTTGTGTCAT    5:32836520
Lizard          TGTCACAAACTGTCAAATACCCAATTTTCATTCTGGTATAAAATGTTCATTTTCTGACAT    1:32929017
Coelacanth      TGACAGAAACTGTCAAATATCTG-CTTTAATTTTGGTATAAAACGTCCACTTAGTGTCAT    JH126706.1:131488
                ** ** ************* *    *** ***   *******  ** ** *   *  ***
```

```
Mouse           GGCGCCAACTGCCATATGCCAGAATAAAGTTAGTGACCCGCCATAAAAACAG-CAGCCTA    2:113674727
Human           GGCGCCAACCACCATATGCCAGAATAAAGTTAGTGACCAGCCATAAAAACAG-CAGCATA    15:33111649
Chimpanzee      GGCGCCAACCACCATATGCCAGAATAAAGTTAGTGACCAGCCATAAAAACAG-CAGCATA    15:29589417
Dog             GGCGCCAACAGCCATATGCCAGAATAAAGTTAGTGACCAGCCATAAAAACAG-CAGCATA    30:5129594
Bovine          GGCGCCAGCCGCCATATGCCAGAATAAAGTTAGTGACCAGCCATAAAAACAG-CAGCATA    10:29908516
Opossum         GGCGCCAATGGCCATATGCCGGAATAAAGTCAGTGACTAGCAATAAAAGGA---AATTCA    1:188909758
Chicken         GGCGCCAACCACCATATGCTGGAATAAAGTCGGTAACCAGCAATAAAAAAAG-CAACATA    5:32836579
Lizard          GGCGCCAACCACCATATGCCAGAATAAAGTCAGTAAGCAGCAATAAAAAAAGGCAACATA    1:32928957
Coelacanth      GGCGCCAACCACCATATGCCGGAATAGAGCCAGTAAACTGCAATAAAAAAAG--AACTTA    JH126706.1:131546
                *******    ********  ***** **   ** *   ** ******  *   *    *

Mouse           GGCACCTTTGAAACCATACAA-GTCATCATGGGTGAGAACGA-GCCATATAAATCCA-TG    2:113674784
Human           AGCACCTTTGAAACGGTACAA-GTCATCGTGGGTGAGAACGA-CCCATATAAATCCA-TA    15:33111603
Chimpanzee      AGCACCTTTGAAACGGTACAA-GTCATCGTGGGTGAGAACGA-CCCATATAAATCCA-TA    15:29589360
Dog             AGCACCTTTGAAATGGTGCAA-GTCATCGTGGGTGAGAACGA-GCCATATAAATCCA-TG    30:5129651
Bovine          AGCACCTTTGAAACGGGACAA-GTCATCGTGGGTGAGAACGA-CCCTTATAAATCCA-TG    10:29908573
Opossum         ACCACCTTTGAAACCTTACAA-GCAGTCAGTGGTGAGAAAGA-TGCATATAAACCTT-TG    1:188909815
Chicken         CGCACCTTTGAAACAGTACAA-GCAATCAACAGTGAGAAAGG-TCTATATAAATCTA-TG    5:32836636
Lizard          AGCACCTTTGAAATGGTACAAAGCAATTAACAGTGGGAAAAAGTCTATATAAATCTA-TG    1:32928898
Coelacanth      AGCACCTTTGAAATAGTACAA-GCAATCAGCAGTGGAAAGGG-TATGTGTAAATCTAACG    JH126706.1:131604
                  ***********     *** *   *     ***  **        * **** *

Mouse           GGGAATTAATCTGGGCTTTGCTACTATCCTGCTGAGCATTTACAATGAGCTTCTACTCTG    2:113674844
Human           GGGAATTAATCTTGGCTTTGCTACTATCCTGCTGGGCATTTACAACAAGCTTCTACTCCC    15:33111535
Chimpanzee      GGGAATTAATCTTGGCTTTGCTACTATCCTGCTGGGCATTTACAACAAGCTTCTACTCCC    15:29589300
Dog             GGGAATTAATCTCGGCTTTGCTACTATCCTGCTGTGCATTTACAAT--------------    30:5129697
Bovine          GGGAATTAATCTCGGCTTTGCTACTATCCTGCTGCGCATTTACAATGAGCTTCCACTCCC    10:29908633
Opossum         GAGAAT-AATTTCAGTTTTGCCACTATCCTACTGGGCATTTACAATGAACCTC-------    1:188909867
Chicken         GAGAATTAATCTCAGCTTTGCCACTATCCTGCTGTGCATTTACAATGAGATTTTGCTCCC    5:32836696
Lizard          GAGAATTAATCTCAGGTTTGCCACTATCCTGCTGCGTATTTACAATGAGCCTCTGCTCCC    1:32928838
Coelacanth      GAGAATTGATCTCAGGTTTGCCACCAACCTGCTGGGTGCCTATAATGAGTCTCTGCTCTG    JH126706.1:131664
                * ****  ** *  * ***** ** * *** *** *    ** **
```

HMCO3 conserved core

```
Mouse           A-----------------CTCTAAACTATTTCTGATCTTCATC-TTTATTTT-CAGACAA    2:113618954
Human           A-----------------CTCTAAACTGTTTCTGATATTCAAC-TCTATTTT-CAAACAC    15:33165537
Chimpanzee      A-----------------CTCTAAACTGTTTCTGATCTTCAAC-TCTATTTT-CAAACAC    15:29643510
Dog             AAGA---------AATAGCTCTAAACTATTTCTGAATTTTAGC-TTCATTTT-CAGACAT    30:5079021
Bovine          ----------------------------------------AGC-TTTATTTT-CAGACAC    10:29837054
Opossum         -------------------------------------------------TTT-CAGAAAG    1:188837695
Chicken         ------------------------------------------------------------
Lizard          A-----------------CACTGAACTATTTCTAGTGTT--ACATTTATTTT-AAAAATG    1:32940147
Coelacanth      AAAAGCAGTGTTGAAATTTTCCAGGTTGTACTTGGTCTTTCTCATTTGCTTTACAGTATG    JH126706.1:97666


Mouse           ATCCTGAGAAACCCTGACTCCCAGCGAATTGACAATGTGGCCCTCTTTATG-TGAAGCCT    2:113619013
Human           ATCCTAAGAAATCCCTACTCCCCCAAAATTGACAATGCAGCCTTCTTTATA-TGAAACCT    15:33165478
Chimpanzee      ATCCTAAGAAATCCCTACTCCCCCAAAATTGACAATGCAGCCTTCTTTATA-TGAAACCT    15:29643451
Dog             ATTCTGAGAAGTCCCAACTCTTAGTGAACTGACAGCACAGCCCTCTTTATA-TGAAGCCT    30:5079080
Bovine          ATCCTAAGAAATCTTGACTCCCAGCAAATTGACAATACAGCCTTCTTTATA-TGAAGCCT    10:29837113
Opossum         GTCTTCAGAAATATTGGCTTCCAACTGATTGACAGTGCAGTCTTATTTTTGACAAAGTCT    1:188837754
Chicken         ---------AATCCAGTCT-------GATTGACACTGAAATGCTCTTTACA-TGAAAGTT    5:32819795
Lizard          ACATTTAAAAATACCAACTCCTACCTGGCAGACATTGCAGTACTACTTGCA-TGAAGGTT    1:32940088
Coelacanth      GTCC--ATAAAGACCAATTCCTGCTTGTTAGATACCATAGGCCCTTTAATG-TGGTGACT    JH126706.1:97723
                         *        *           ** *            *            *

Mouse           TGTTTATAGGCCGCAATTCTCCTGACAGATTTACAAGAGTCTGTGGAGTTTTAAAGGACG    2:113619073
Human           TGTTTATAGGCCACAATTCTCCCGACAGATTTACAAGTGTCTGTGGAGTTTTAAAAGACG    15:33165418
Chimpanzee      TGTTTATAGGCCACAATTCTCCCGACAGATTTACAAGTGTCTGTGGAGTTTTAAAAGACG    15:29643391
Dog             TGTTTATAGGCCACAATTCTCCCGACAGATTTACAAGTGTCTGTGGAGTTTTAAAAGACG    30:5079140
Bovine          TGTTTATAGGCTACAATTCTCCCGACAGATTTACAAGTGTCTGCGGAGTTTTAAAAGATG    10:29837173
Opossum         TGTTTATAAACCTCAATTCTCCTGACAGATTTACAAGTATCTGTGGAGTTTTAA--GACG    1:188837812
Chicken         TGTTTATGGGCAGTGATTCTTTTGACAGATTTACAAGTGCCTGTGGAGTTTTAAAAGACT    5:32819855
Lizard          TGTTTATACATAATGATTCTTTAGACAGATTTACAAGTGCCTGTGGAGTTTTAAAAGACT    1:32940028
Coelacanth      TAACCATAGGTGATGACGCCAATGACAGGTTTACAAGTGTCCATGGTATTTTAAAAGATG    JH126706.1:97783
                *    **        *  *    ***** ********   *   **  ******  **
```

```
Mouse           ATTGCTTAACAAGCATTAAGTGAG------------------------------------    2:113619097
Human           ATTGCTTAACAGGCATTAAGTGAG------------------------------------    15:33165394
Chimpanzee      ATTGCTTAACAGGCATTAAGTGAG------------------------------------    15:29643367
Dog             ATTGCTTAACAGGCATTAAGTGAG------------------------------------    30:5079164
Bovine          ATTGCTTAACCGGCATTAAGTGAG------------------------------------    10:29837197
Opossum         GTTGCCTAATAGGCATTAAGTGAG------------------------------------    1:188837836
Chicken         ATTGCCCAATAGGCATTAAGTGAG------------------------------------    5:32819879
Lizard          ATTGCCCAACAGGCATTAAGGAAG------------------------------------    1:32940004
Coelacanth      CTTAGCCAATAGATATCTAGTGAATTTGTGTGTAATAAATGAACTAATAAACTACAGCTG    JH126706.1:97843
                 **    **     **  **  *
```
