# Supplementary material for: Conserved cis-regulatory regions in a large genomic landscape control SHH and BMP-regulated Gremlin1 expression in mouse limb buds
Source: BMC Dev Biol. 2012 Aug 13;12:23. doi: 10.1186/1471-213X-12-23 (PMC3541112; doi:10.1186/1471-213X-12-23)
Supplement: Additional file 2 — Table S1. Genomic coordinates for the sequence comparisons shown in Figure 1A. [file 1471-213X-12-23-S2.docx]

**Table S1**

**Genomic coordinates for the sequence comparisons shown in Fig. 1A**

| *Region* | *Species* | *% ID (threshold 74%)* | *Length (100bp threshold)* | *Coordinates* |
| --- | --- | --- | --- | --- |
| HMCO1 | Zebrafish | - | - | - |
| HMCO1 | Xenopus | 74.7% | 100 | xenTro2 scaffold_37:3687354-3687453 |
| HMCO1 | Chicken | 80.2% | 381 | galGal3 chr5:32849567-32849947 |
| HMCO1 | Opossum | 81.3% | 430 | monDom5 chr1:188935147-188935576 |
| HMCO1 | Dog | 80.5% | 387 | canFam2 chr30:5150126-5150512 |
| HMCO1 | Chimpanzee | 81.5% | 508 | panTro3 chr15:29568244-29568751 |
| HMCO1 | Human | 81.5% | 508 | hg19 chr15:33090437-33090944 |
| HMCO2 | Zebrafish | - | - | - |
| HMCO2 | Xenopus | - | - | - |
| HMCO2 | Chicken | 80.4% | 377 | galGal3 chr5:32836351-32836727 |
| HMCO2 | Opossum | 80.1% | 380 | monDom5 chr1:188909506-188909885 |
| HMCO2 | Dog | 84.2% | 494 | canFam2 chr30:5129238-5129731 |
| HMCO2 | Chimpanzee | 84.6% | 818 | panTro3 chr15:29588916-29589733 |
| HMCO2 | Human | 84.5% | 818 | hg19 chr15:33111145-33111962 |
| HMCO3 | Zebrafish | - | - | - |
| HMCO3 | Xenopus | - | - | - |
| HMCO3 | Chicken | 74% | 272 | galGal3 chr5:32819753-32820024 |
| HMCO3 | Opossum | 77% | 331 | monDom5 chr1:188837685-188838015 |
| HMCO3 | Dog | 78.3% | 842 | canFam2 chr30:5078806-5079647 |
| HMCO3 | Chimpanzee | 80.7% | 940 | panTro3 chr15:29642824-29643763 |
| HMCO3 | Human | 80.1% | 937 | hg19 chr15:33164854-33165790 |
