# Supplementary material for: Conserved cis-regulatory regions in a large genomic landscape control SHH and BMP-regulated Gremlin1 expression in mouse limb buds
Source: BMC Dev Biol. 2012 Aug 13;12:23. doi: 10.1186/1471-213X-12-23 (PMC3541112; doi:10.1186/1471-213X-12-23)
Supplement: Additional file 3 — Table S2. Analysis of transgenic founder embryo. [file 1471-213X-12-23-S3.docx]

**Table S2**

**Analysis of transgenic founder embryo**

| Transgene | Embryos * | Forelimb bud expression | Figure |
| --- | --- | --- | --- |
| *Grem1-LacZ* | 3 | **3 posterior** | **Fig.1B** |
| *Grem1-LacZ ∆HMCO1* | 7 | 1 strong  1 very weak  **5 no expression** | **Fig.1C** |
| *Grem1-LacZ ∆HMCO2* | 3 | **2 weak posterior**  1 no expression | **Fig.1D** |
| *Grem1-LacZ ∆HMCO3* | 5 | **3 posterior**  2 no expression | **Fig.1E** |
| HMCO123-*ßglob-LacZ* | 3 | **3 anterior** | **Fig.2A** |
| *GRS1-ßglob-LacZ* | 2 | **2 posterior** | **Fig.2B** |

* Founder embryos with ß-galactosidase activity anywhere in limb buds and/or trunk
