# Supplementary figures and images for: Conserved cis-regulatory regions in a large genomic landscape control SHH and BMP-regulated Gremlin1 expression in mouse limb buds
Source: BMC Dev Biol. 2012 Aug 13;12:23. doi: 10.1186/1471-213X-12-23 (PMC3541112; doi:10.1186/1471-213X-12-23)

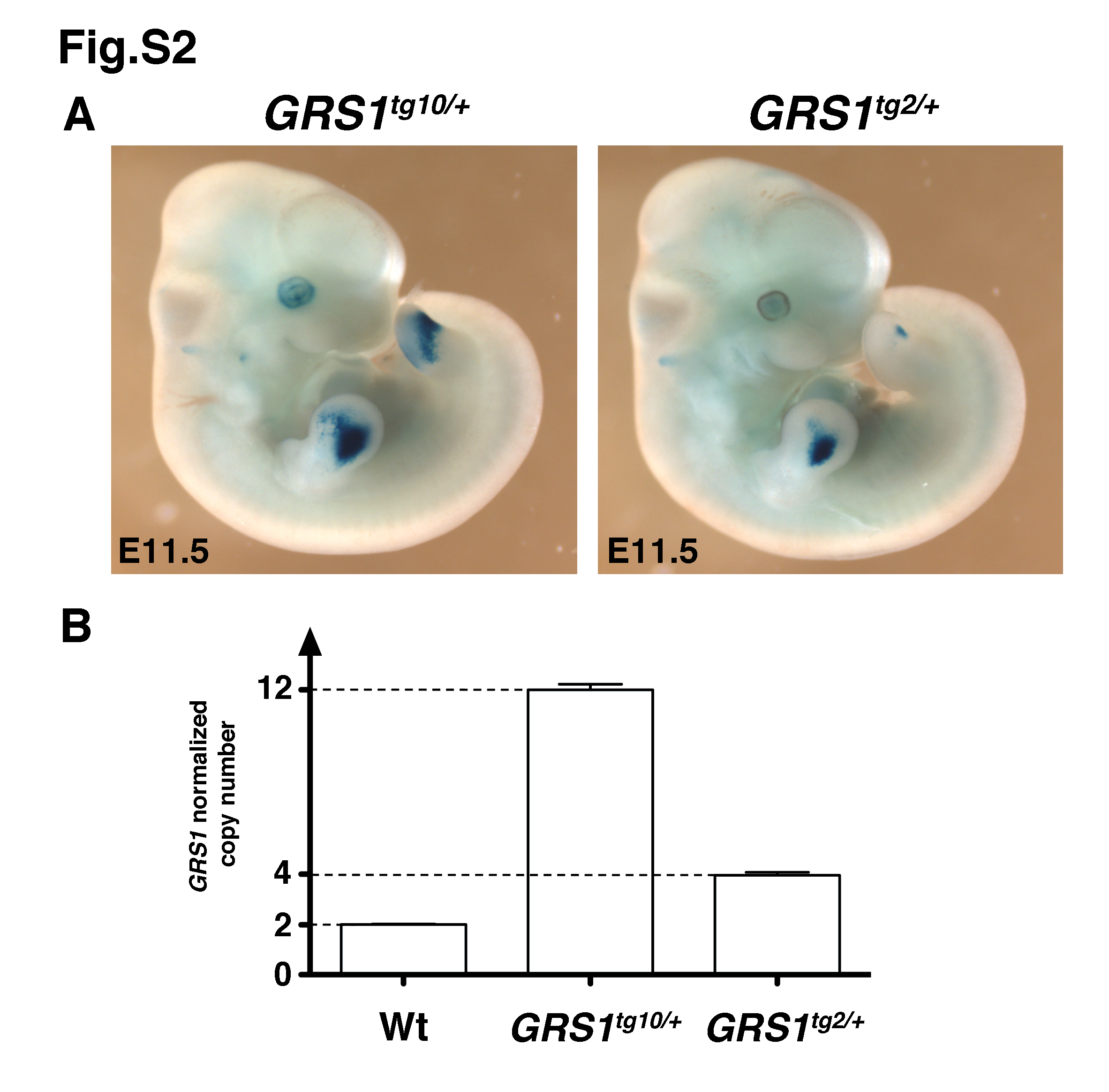

Supplement: Additional file 4 — Figure S2. Limb bud mesenchymal expression of GRS1-ßglob-LacZ transgene in two independent transgenic mouse strains. (A) Expression of the GRS1-ßglob-LacZ transgene is restricted to limb buds, with expression initiating earlier in forelimb than hindlimb buds. Note the differences in ß-galactosidase activity in GRS1tg10/+ (left panel) and GRS1tg2/+ (right panel) transgenic mouse embryos. (B) Using real-time qPCR, the transgene copy numbers in both the GRS1tg10/+ and GRS1tg2/+ mouse strains were determined in comparison to wild-type mice (carrying 2 copies of the endogenous GRS1 regions). This analysis revealed that the GRS1tg10/+ strain carries 10 copies and GRS1tg2/+ 2 copies of the transgene, respectively. [file 1471-213X-12-23-S4.tiff]

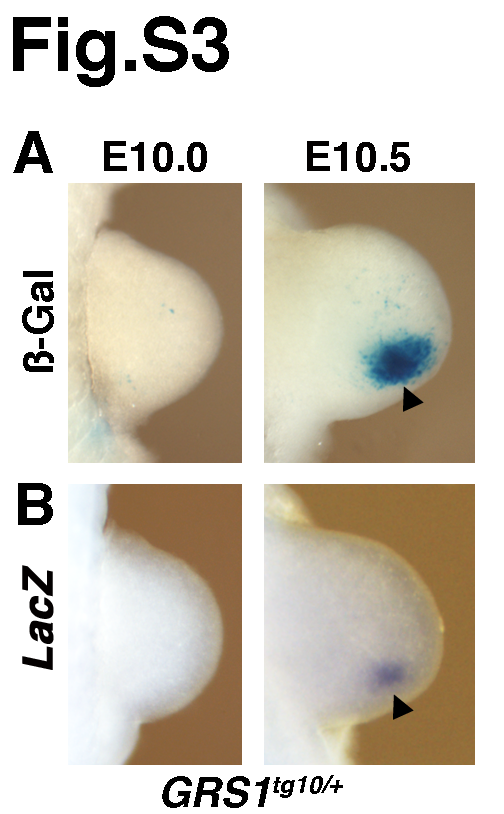

Supplement: Additional file 5 — Figure S3. Comparison of the LacZ mRNA and ß-galactosidase reporter activity in early limb buds of GRS1tg10/+ embryos. Distribution of ß-galactosidase activity (A) and LacZ transcripts (B) in forelimb buds of GRS1tg10/+ embryos at E10.0 and E10.5. Arrowheads point to the posterior expression domains. [file 1471-213X-12-23-S5.tiff]

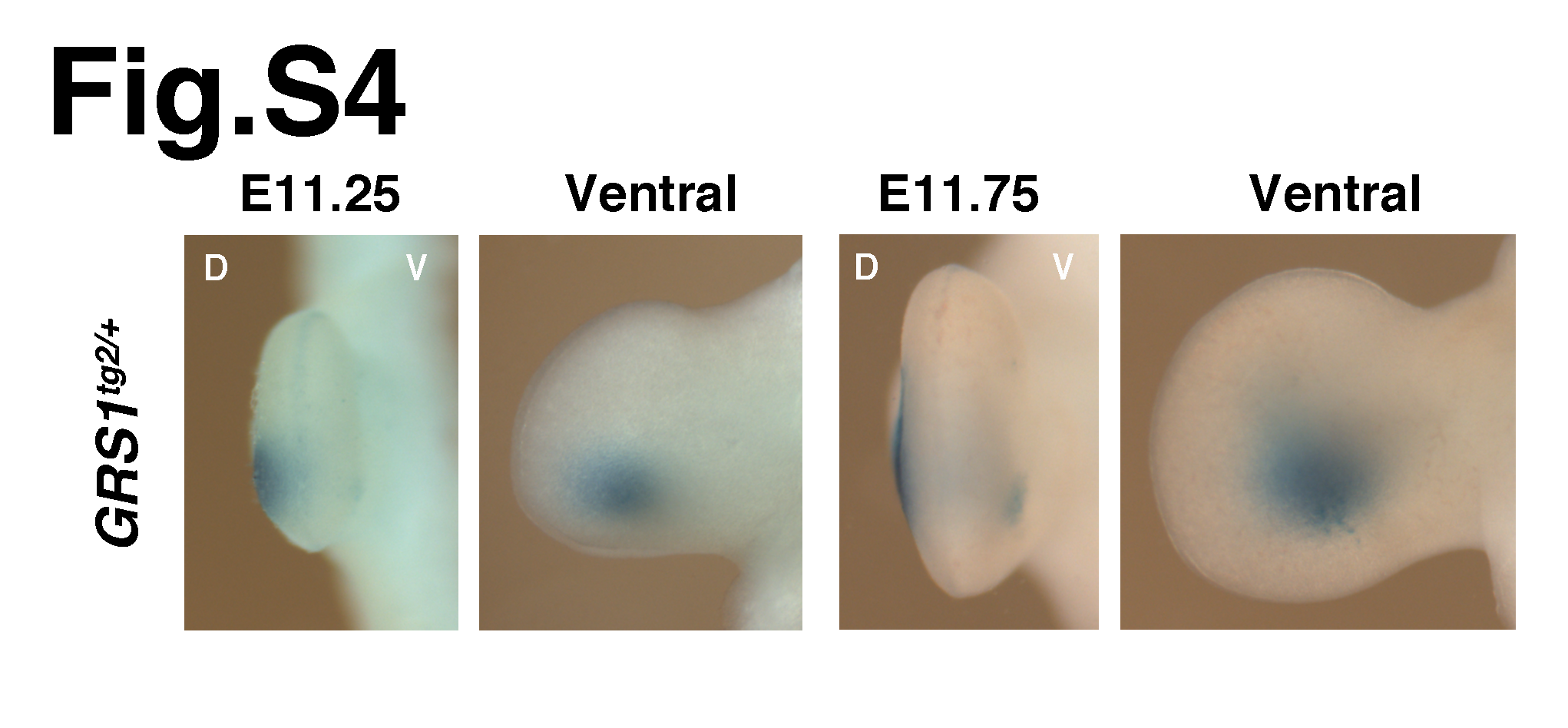

Supplement: Additional file 6 — Figure S4. Dorso-ventral distribution of ß-galactosidase activity in forelimb buds expressing the GRS1tg2/+ transgene. The GRS1tg2/+ transgene respects the dorsal and ventral restriction of the mesenchymal expression domains. Note that overall expression is significantly lower than in GRS1tg10/+ limb buds (compare to Figure 5B). D: dorsal, V: ventral. [file 1471-213X-12-23-S6.tiff]
