# Supplementary material for: Conserved cis-regulatory regions in a large genomic landscape control SHH and BMP-regulated Gremlin1 expression in mouse limb buds
Source: BMC Dev Biol. 2012 Aug 13;12:23. doi: 10.1186/1471-213X-12-23 (PMC3541112; doi:10.1186/1471-213X-12-23)
Supplement: Additional file 7 — Table S3. qPCR amplicons for GLI3 ChIP analysis. [file 1471-213X-12-23-S7.docx]

**Table S3**

**qPCR amplicons for GLI3 ChIP analysis**

| *GLI3 ChIP amplicon* | *Coordinates (mm10)* |
| --- | --- |
| a | chr2:113691635-113691714 |
| b | chr2:113692244-113692320 |
| c | chr2:113693100-113693179 |
| d | chr2:113693471-113693578 |
| e | chr2:113697427-113697518 |
| f | chr2:113674650-113674732 |
| g | chr2:113675141-113675273 |
| h | chr2:113618703-113618814 |
| i | chr2:113619171-113619319 |
