# Supplementary material for: Conserved cis-regulatory regions in a large genomic landscape control SHH and BMP-regulated Gremlin1 expression in mouse limb buds
Source: BMC Dev Biol. 2012 Aug 13;12:23. doi: 10.1186/1471-213X-12-23 (PMC3541112; doi:10.1186/1471-213X-12-23)
Supplement: Additional file 8 — Table S4. Genomic coordinates of identified Grem1 regulatory regions. [file 1471-213X-12-23-S8.docx]

**Table S4**

**Genomic coordinates of identified *Grem1* regulatory regions**

| *Annotation* | *Length* | *Coordinates (mm10)* |
| --- | --- | --- |
| HMCO1 | 520 bp | chr2:113693204-113693723 |
| HMCO2 | 1279 bp | chr2:113674096-113675374 |
| HMCO3 | 924 bp | chr2:113618678-113619601 |
| *GRS1-ßglob-LacZ* transgene | 9’831 bp | chr2:113689791-113699621 |
| GBR1 (HMCO1, ChIP amplicons c-d) [[32](#_ENREF_32)] | 439 bp | chr2:113693005-113693989 |
| GBR2 (ChIP amplicons a-b) [[32](#_ENREF_32)] | 700 bp | chr2:113691653-113692352 |
| GBR3 (HMCO2, ChIP amplicons f-g) [[32](#_ENREF_32)] | 985 bp | chr2:113674455-113675313 |
| GBR4 (with GLI binding motif) [[32](#_ENREF_32)] | 439 bp | chr2:113640843-113641281 |
| CTCF binding region [[31](#_ENREF_31)] | 468 bp | chr2:113648107-113648574 |
